# Development of a practical approach to expert elicitation for randomised controlled trials with missing health outcomes: Application to the IMPROVE trial

**DOI:** 10.1177/1740774517711442

**Published:** 2017-07-04

**Authors:** Alexina J Mason, Manuel Gomes, Richard Grieve, Pinar Ulug, Janet T Powell, James Carpenter

**Affiliations:** 1 Department of Health Services Research and Policy, London School of Hygiene & Tropical Medicine, London, UK; 2 Vascular Surgery Research Group, Imperial College London, London, UK; 3 Department of Medical Statistics, London School of Hygiene & Tropical Medicine, London, UK

**Keywords:** Missing data, sensitivity analysis, expert elicitation, Bayesian analysis, clinical trials, pattern-mixture models, quality of life

## Abstract

**Background/aims:**

The analyses of randomised controlled trials with missing data typically assume that, after conditioning on the observed data, the probability of missing data does not depend on the patient’s outcome, and so the data are ‘missing at random’ . This assumption is usually implausible, for example, because patients in relatively poor health may be more likely to drop out. Methodological guidelines recommend that trials require sensitivity analysis, which is best informed by elicited expert opinion, to assess whether conclusions are robust to alternative assumptions about the missing data. A major barrier to implementing these methods in practice is the lack of relevant practical tools for eliciting expert opinion. We develop a new practical tool for eliciting expert opinion and demonstrate its use for randomised controlled trials with missing data.

**Methods:**

We develop and illustrate our approach for eliciting expert opinion with the IMPROVE trial (ISRCTN 48334791), an ongoing multi-centre randomised controlled trial which compares an emergency endovascular strategy versus open repair for patients with ruptured abdominal aortic aneurysm. In the IMPROVE trial at 3 months post-randomisation, 21% of surviving patients did not complete health-related quality of life questionnaires (assessed by EQ-5D-3L). We address this problem by developing a web-based tool that provides a practical approach for eliciting expert opinion about quality of life differences between patients with missing versus complete data. We show how this expert opinion can define informative priors within a fully Bayesian framework to perform sensitivity analyses that allow the missing data to depend upon unobserved patient characteristics.

**Results:**

A total of 26 experts, of 46 asked to participate, completed the elicitation exercise. The elicited quality of life scores were lower on average for the patients with missing versus complete data, but there was considerable uncertainty in these elicited values. The missing at random analysis found that patients randomised to the emergency endovascular strategy versus open repair had higher average (95% credible interval) quality of life scores of 0.062 (−0.005 to 0.130). Our sensitivity analysis that used the elicited expert information as pooled priors found that the gain in average quality of life for the emergency endovascular strategy versus open repair was 0.076 (−0.054 to 0.198).

**Conclusion:**

We provide and exemplify a practical tool for eliciting the expert opinion required by recommended approaches to the sensitivity analyses of randomised controlled trials. We show how this approach allows the trial analysis to fully recognise the uncertainty that arises from making alternative, plausible assumptions about the reasons for missing data. This tool can be widely used in the design, analysis and interpretation of future trials, and to facilitate this, materials are available for download.

## Introduction

In randomised controlled trials (RCTs), outcome data are typically missing for some participants. Patient-reported outcomes such as health-related quality of life (QoL) are particularly prone to missing data because patients may fail to complete follow-up questionnaires.^1,2^ Missing data can reduce the power and efficiency of an RCT and also lead to biased effectiveness estimates.^3–6^ In the primary trial analysis, studies are recommended to take an approach that is valid under plausible assumptions about the missing data.^7^ Rather than assuming that the data are ‘missing completely at random’ (MCAR), the primary analysis should assume they are ‘missing at random’ (MAR), that is, the probability of missing data does not depend on the patient’s outcome, after conditioning on the observed variables (e.g. the patients baseline characteristics). However, the MAR assumption may be implausible in many settings; for example, patients in relatively poor health may be less likely to complete the requisite questionnaires, and so these outcome data may be ‘missing not at random’ (MNAR). As the true missing data mechanism is unknown given the data at hand, it is important to examine whether the study results are robust to alternative assumptions about the missing data.

The US National Research Council (NRC) report on missing data in clinical trials recommended sensitivity analyses that recognised the data could be MNAR,^8^ in line with general methodological guidance for dealing with missing data,^9^ and previous specific advice for intention-to-treat analysis in RCTs.^6^ However, systematic reviews report that in practice RCTs do not handle missing data appropriately.^10,11^

A simple approach to sensitivity analysis is to include in the statistical model parameters representing outcome differences between individuals with complete versus missing data and explore how inference vary as these ‘sensitivity parameters’ take on specific values.^12^ The results and conclusions can then be compared over a plausible range of values, possibly including a ‘tipping-point’ at which the results change. However, drawbacks of this approach are (1) for each sensitivity analysis the sensitivity parameters are treated as known, without uncertainty; (2) the challenge of determining what constitutes a plausible range, and relative difficulty of making statistical model parameters accessible to non-statistical experts; (3) the extent to which some values should be considered more plausible; and (4) the difficulty that the plausibility of parameters/tipping points are often assessed after the experts have seen the preliminary analyses. An alternative is to allow experts to quantify their views, rather than those of others. Not only is this likely to be more intuitive and attractive for them, but (as we show in this article) it allows us to take a fully Bayesian approach and properly capture and reflect expert opinion (and associated uncertainty) about the missing data in the posterior estimate of the treatment effect and its credible interval.

This is particularly useful for those needing a quantitative summary of the trial, such as systematic reviewers, decision makers and health providers, because it provides a quantitative summary of how those involved in the study (experts) would interpret its results given the missing data. When reviewing the study, experts will automatically (implicitly) ‘fill in’ the gaps created by the missing data to arrive at their conclusions. The proposed elicitation approach coupled with a Bayesian analysis allows the study to coherently quantify the impact of incorporating expert knowledge about the missing data, through to the estimates of treatment effectiveness.

The Bayesian approach allows uncertainty about the missing data mechanism to be propagated through the eventual estimates of relative effectiveness. Such sensitivity analyses require practical tools to facilitate expert elicitation, and recent research, for example, the Sheffield Elicitation Framework and associated web-based elicitation tool,^13,14^ has focused on elicitation approaches within group meetings. As Hampson et al.^15^ illustrate, group-level elicitation has advantages for training and clarification and facilitates behavioural aggregation, such as Delphi processes, for achieving consensus.^16^ However, because of the ‘feedback’ loop, these approaches are costly in both money and time, and in many RCTs, it may be infeasible to elicit opinion from a sufficient number and range of experts. To improve the uptake of recommended approaches to sensitivity analysis for missing data within RCTs requires that more accessible, practical tools for eliciting and synthesising expert opinion are developed and exemplified.^8^

This article directly addresses this gap in the literature, by developing a practical elicitation tool for eliciting the expert opinion required for sensitivity analysis that allows for data to be MNAR. The tool can quickly elicit views from tens of experts, who have limited time to devote to the elicitation exercise. We illustrate our elicitation tool with the motivating example of Immediate Management of the Patient with Rupture: Open Versus Endovascular strategies (IMPROVE), an ongoing multi-centre trial with a parallel design which evaluates the effectiveness of an emergency endovascular strategy (eEVAR) compared with open repair (OPEN) for patients with ruptured abdominal aortic aneurysm (ISRCTN 48334791, www.improvetrial.org). In the IMPROVE trial, 21% of patients did not complete follow-up EQ-5D-3L questionnaires at 3 months post-randomisation.

The article proceeds as follows: section ‘Motivating study: the IMPROVE trial’ outlines the IMPROVE trial and the requirements for the elicitation exercise. Sections ‘Development of the elicitation tool’ and ‘Eliciting and synthesising expert opinion’ explain how the elicitation tool was developed and used. Section ‘Results’ gives the results. Section ‘Discussion’ discusses the findings in the context of related research and outlines areas for further research.

## Motivating study: the IMPROVE trial

The IMPROVE trial recruited 613 patients from 30 sites (29 in the United Kingdom, 1 in Canada). The published analyses found that there was no difference in the primary endpoint of 30-day mortality between the randomised arms,^17^ but that patients with ruptured aneurysms who were randomised to the eEVAR strategy had on average, a clinically significant improvement in their QoL score at both 3 and 12 months versus those randomised to open repair.^18^ The QoL assessment used the EQ-5D-3L^19^ questionnaire which requires patients to describe their own health according to five dimensions: mobility, self-care, usual activities, pain or discomfort and anxiety or depression, with the option of three levels of severity: ‘no problems’, ‘some problems’ and ‘extreme problems’. The responses to these questions are then combined with preference values from the published literature,^20^ to provide QoL index scores on a scale anchored at 1 (perfect health) and 0 (death) with health states judged worse than death assigned a negative value. The published analyses used multiple imputation to handle the missing data assuming MAR, so it is unclear whether the reported gain in average QoL for the eEVAR strategy is robust to plausible departures from MAR.

We use expert elicitation to recognise that the follow-up QoL data in IMPROVE may be MNAR. At 3 months, eligible patients in both randomised arms failed to return completed QoL questionnaires. As it is anticipated that the patients’ response to treatment will differ, for example, hospital stay will be longer for the open repair arm compared to the eEVAR arm, it is plausible that the reasons for the missingness and associated missing values also differ by intervention. Therefore, we elicited expert beliefs about expected QoL differences between patients with missing versus fully observed QoL data for patients in each arm. Our elicitation was restricted to eligible survivors, that is, those with confirmed ruptured aneurysms who had survived up to 3 months.^18^ If differences in mortality rates between the two arms had been found, these would feed through into quality-adjusted life years, but there would be no implications for the elicitation or analysis method.

### Ethical approval for this study

Ethical approval was given by the London School of Hygiene and Tropical Medicine observational research ethics committee. Also, this study was approved by the IMPROVE Trial Management Committee.

### Pattern-mixture model

Missing data that are MNAR may be modelled with selection models or pattern-mixture models.^9^ To encourage uptake of these sensitivity analyses in RCTs, we adopt a pattern-mixture approach. This is because in our dealings with regulators and trial statisticians in academia and industry, we have found the underlying assumptions are more accessible to those interpreting the results of RCTs. Consistent with the published analysis, we undertake an intention-to-treat analysis and estimate the effect of randomised arm on QoL at 3 months for eligible patients.

**Figure 1.**
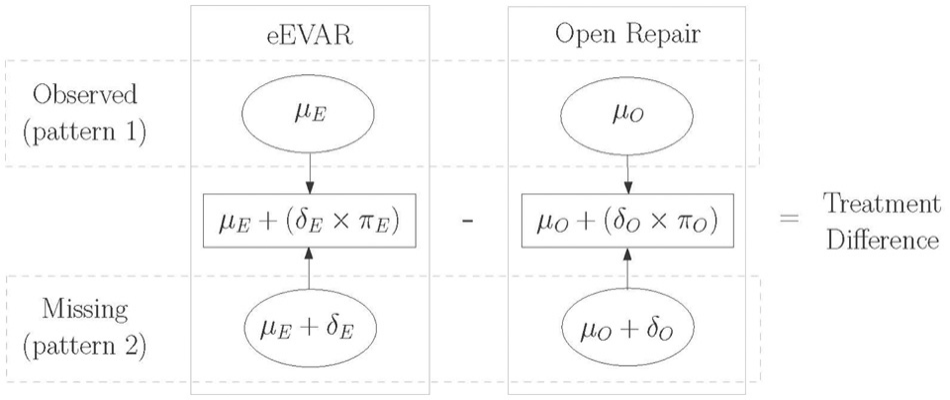
Illustration of the estimation of treatment effectiveness using a pattern-mixture model that allows for outcome data to be MNAR. μ represents the mean QoL for patients who returned their QoL questionnaires, δ represents the difference in the mean QoL between patients who did and did not return their QoL questionnaires and π represents the proportion of patients who did not return their QoL questionnaires. *E* and *O* indicate the eEVAR and open repair treatment groups respectively. **Simple arithmetic example that uses hypothetical elicited values to re-calculate the effectiveness of eEVAR versus open repair on QoL score. The example uses a pattern-mixture model to allow for data that are MNAR**. Information from QoL data that are observed in the RCT: sample mean (SE) QoL score for patients who completed QoL questionnaire eEVAR strategy=0.76(0.02),open repair strategy=0.69(0.03) proportion of patients who did not return their QoL questionnaire eEVAR strategy=0.18,open repair strategy=0.24 Information elicited from an expert: mean (SD) of difference in mean QoL between patients who did and did not return their QoL questionnaire eEVAR strategy=−0.01(0.04),open repair strategy=−0.05(0.1) Then a point estimate of the treatment difference can be calculated as $(\mu_{E}+\pi_{E}\delta_{E})-(\mu_{O}+\pi_{O}\delta_{O})=(0.76-0.18\times 0.01)-(0.69-0.24\times 0.05)=0.08.$ Assuming independence between variables, the variance (*V*) of the treatment difference is $V(\mu_{E})+\pi_{E}^{2}V(\delta_{E})+V(\mu_{O})+\pi_{O}^{2}V(\delta_{O})=0.02^{2}+0.18^{2}0.04^{2}+0.03^{2}+0.24^{2}0.1^{2}=0.002,$ and a 95% confidence interval (CI) for the treatment difference can be estimated as (0.08−1.96×0.044,0.08+1.96×0.044)=(−0.01,0.17) Hence using a pattern-mixture model with expert information reports an estimate of the effectiveness of eEVAR versus open repair of treatment difference (95% CI) of 0.08 (−0.01, 0.17), compared to 0.07 (0.00, 0.14) for calculations based on the observed QoL alone. Note the wider confidence interval from using the pattern-mixture model, as this approach takes account of the uncertainty from the missing data that may be MNAR.

In our example, the pattern-mixture model allows the mean QoL to be calculated differently according to whether the QoL is observed (pattern 1) or missing (pattern 2). For pattern 1, we can calculate the mean response for each arm from the observed data (μ). However, for pattern 2, the outcome data are missing, and so we calculate the mean to be that for pattern 1 plus an offset (δ). As shown in Figure 1, the effectiveness of treatment can then be estimated by weighting the mean QoL scores in each pattern using the proportion of patients with missing data in each arm (π). The offset term, also known as a sensitivity parameter, may well differ according to prognostic factors. So in the IMPROVE trial, $\delta_{O}$ and $\delta_{E}$ represent the difference in QoL between those who did and did not complete the questionnaire for the eEVAR and OPEN arms, respectively. A key concern is that they cannot be estimated from the observed data.

### What information is required?

To estimate treatment effectiveness, recognising that data may be MNAR, we require expert opinion about the likely values of the difference in the mean QoL between patients who did and did not complete the QoL assessment. This comparison will be made for patients who are similar according to characteristics that we have observed, such as age, gender and baseline disease severity. We will also make this comparison to estimate this sensitivity parameter for each randomised arm ($\delta_{O}$ and $\delta_{E}$). To see how this might work, suppose that an expert’s views can be summarised by a mean which gives their most likely value and a standard deviation, which represents their uncertainty about this value. Then, as we show in Figure 1, we could simply substitute the mean values into the formula to provide an estimate of treatment effectiveness that reflects this expert’s opinion about the outcome differences between patients with missing versus complete outcome data. In the worked example, the expert expects patients in the open repair arm who did not complete a questionnaire to have a lower QoL score than those who did. The net effect of including this elicited value for this sensitivity parameter is that the average effectiveness of eEVAR is somewhat larger than in the MAR analysis. As the worked example shows, the expert’s uncertainty about the missing values can be propagated through into the estimates of effectiveness. The worked example in Figure 1 uses standard formulae, but we would like to perform a more sophisticated analysis incorporating elicited information from multiple experts, adjusting for observed differences in baseline characteristics and correlations in the QoL scores between the trial arms. Using the same principles, we show how Bayesian methods can provide a practical way of implementing these improvements.

This approach to missing data requires that beliefs are elicited from those experts with knowledge about the likely outcomes of patients who did not complete QoL questionnaires. We identified 46 potential experts, who were principal investigators (mainly consultant vascular surgeons) or trial coordinators (vascular nurse specialists or research nurses), had been in their post for at least 2 years and had ongoing involvement in the IMPROVE trial. These experts were judged likely to have knowledge about the prognosis and outcomes of the trial patients, beyond that recorded in the data.

## Development of the elicitation tool

The main purpose of the elicitation was to quantify differences in the mean QoL score between patients who did and did not complete QoL questionnaires. Specifically, we asked the experts to provide their beliefs about QoL for ‘typical’ IMPROVE trial patients, stressing that these typical patients were similar according to observed characteristics, and the only differences between them were the randomised arm and whether or not they returned a completed QoL questionnaire.

Hogarth^21^ advised that ‘assessment techniques should be designed both to be compatible with man’s abilities and to counteract his deficiencies’. Following this, our work builds on our and others’ previous work (e.g. White et al.,^22^ Mason,^23^ and references therein) suggesting the benefits of a graphical approach. Whereas previous work has elicited quantiles or other summaries from experts and then provided graphical feedback,^24,25^ we made our approach more intuitive and interactive, by allowing the expert to manipulate the distribution directly from the start.

We developed an easy-to-use web-based elicitation tool using Shiny, a web application framework within a widely used statistical software, R,^26,27^ which could be administered by e-mail or in conference breaks. We minimised the administrative burden by collecting informed consent electronically and offered a £20 Amazon gift card as a token of appreciation for completed surveys. ‘Good practice’ recommendations for eliciting expert opinion were followed, in particular by including a feedback question and allowing the experts to revise their answers.^28^

**Figure 2.**
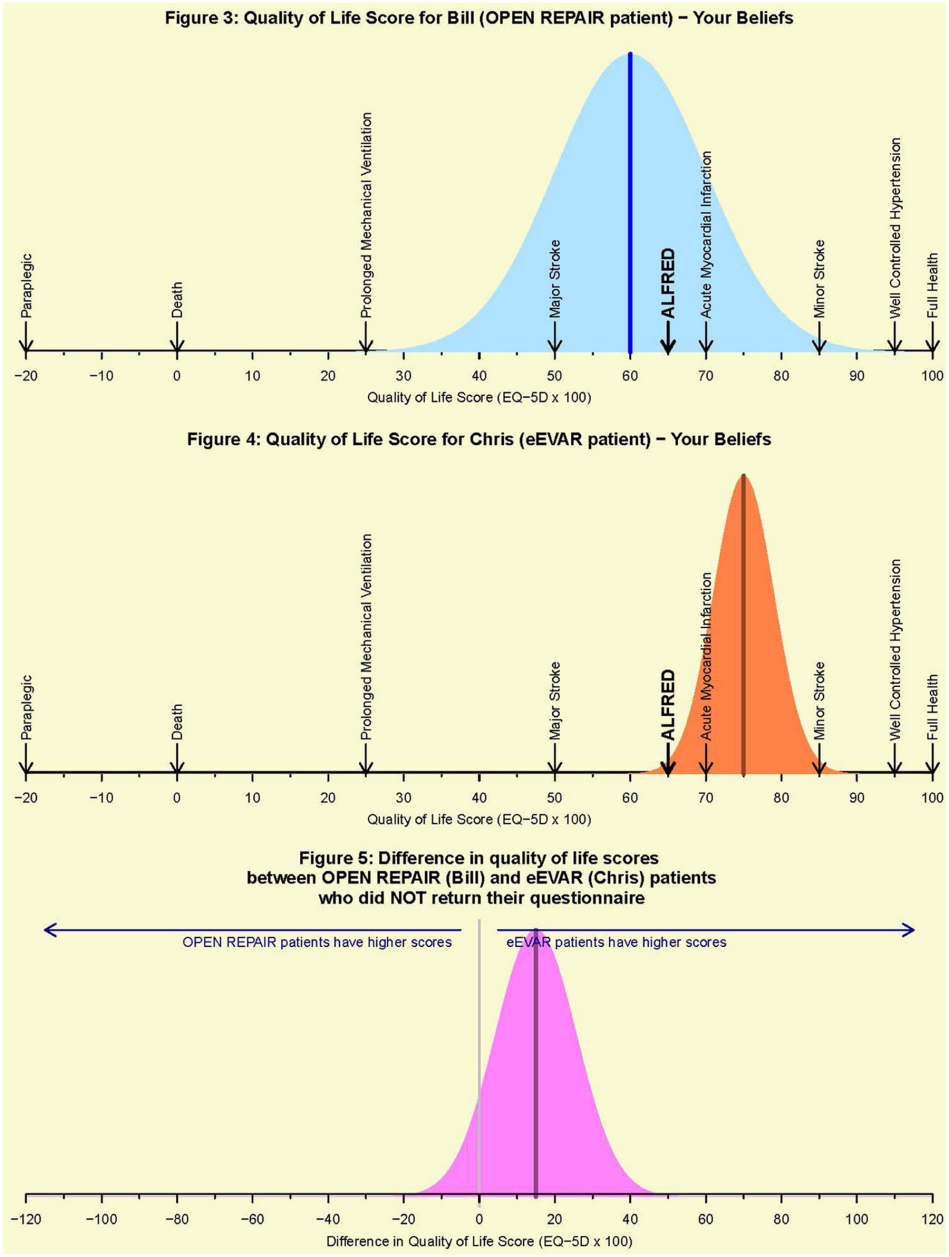
Screen shots from the elicitation tool.

The scale for QoL scores for the elicitation exercise is the same as the original scale for the EQ-5D utility score, multiplied by 100 for ease of completion. The expert is provided with possible QoL scores for typical patients with six exemplar diagnoses on the scale between −20 and 100, chosen as they were anticipated to be familiar to our experts and spanned the QoL scale (see Figure 2). The QoL values for these diagnoses were taken from published literature (see supplementary material for details).

The questionnaire includes some free text questions asking the expert to explain the basis of their views, in terms of what they observed about the trial patients and any other reasons. These provided some useful context which we revisit in the discussion. The supplementary material provides further detail about the elicitation questions.

The tool was pre-piloted to improve usability and accessibility to our target audience, using non-clinical London School of Hygiene and Tropical Medicine clinical trials unit staff with no IMPROVE involvement. Following this, input was provided by the Trial Manager and Chief Investigator. At their suggestion, fictitious cartoon patients (Alfred, Bill and Chris) were incorporated into the elicitation questionnaire, as an aide-memoir of the typical IMPROVE patients.

At the pilot stage, we carried out face-to-face elicitations with four experts, representative of those selected for the main elicitation. They took, on average, 20 min to complete the survey and provided feedback that led to wording changes for improved clarity. However, no major alterations to the structure of the tool were suggested, and the graphical approach with ‘sliders’ received favourable comments.

The final version of the elicitation can be downloaded from https://ajm-elicit.shinyapps.io/ElicitAppHighQ5, and screen shots from some of the key questions are reproduced as Figure 2. The graph in the top panel accompanies the question to elicit likely QoL scores for a typical IMPROVE patient randomised to the open repair strategy who did not complete the questionnaire (Bill). At this stage, the expert has already been introduced to Alfred, a typical patient randomised to the open repair strategy, but who did return a completed QoL questionnaire: Alfred’s score is known and marked on the QoL scale. The expert is asked for the most likely value of Bill’s score and to indicate graphically their uncertainty about this value: the blue curve changes dynamically as the expert moves sliders. The graph in the middle panel is for the corresponding question about a typical patient randomised to the eEVAR strategy who did not complete their questionnaire (Chris). The bottom panel shows the feedback provided to the expert about the implications of their answers in terms of the differences between the QoL scores for patients who did not complete a QoL questionnaire and were assigned to the OPEN (Bill) and eEVAR (Chris) arms respectively, assuming that the elicited distributions for the OPEN and eEVAR arms are not related.

To allow for the possibility that the elicited values in the two arms are related, for example, a high QoL score in the OPEN arm makes a high score in the eEVAR arm more likely, we asked about the score in the eEVAR arm again, but this time provided the score for the OPEN arm (see supplementary material for further details). By eliciting this third distribution, we had sufficient information to formulate a joint prior for the two sensitivity parameters allowing for correlation between them.

## Eliciting and synthesising expert opinion

The chief investigator emailed a participation invitation to all the experts identified as potential respondents, including a web link to the elicitation tool and the participant information sheet. Weekly reminders were sent throughout the following month and we offered a further opportunity to complete the elicitation at The Vascular Society Annual Scientific Meeting in November 2015.

The sensitivity analysis approach required that the uncertainty in the individual responses was recognised and that these responses were then pooled. We first specified individual bivariate normal prior distributions for both of the sensitivity parameters using the responses from each expert. Second, we combined the responses across the experts using linear pooling,^16^ which is a method of mathematical aggregation widely used in practice, calculating an average of the individual distributions using equal weights. This was specified in our Bayesian models as a mixture of the bivariate normal distributions for each expert using the WinBUGS software.^29^ See supplementary material for examples and code.

To fully explore the sensitivity of the trial results to a range of expert opinion, we formed a ‘community’ of priors^30^ comprising three pooled priors (all experts, all doctors and all nurses). To examine the sensitivity of the results to the full range of diversity of opinion, we also considered two individual priors according to the ‘most sceptical’ expert (QoL score 0.2 higher for OPEN) and the ‘most enthusiastic’ expert (QoL score 0.29 higher for eEVAR).

## Results

### Expert responses

Table 1 summarises the characteristics for the 26 experts who completed the survey. Over half of the responses were provided at the conference and almost twice as many doctors as nurses responded.

**Table 1.** Summary of the experts’ characteristics and knowledge of the IMPROVE trial results.

	All		Nurses^a^		Doctors^b^	
Number of responses	26		9		17	
Conference response: n (%^c^)	15	(58%)	6	(67%)	9	(53%)
Years in current role: n (%^c^)						
2–3 years	3	(12%)	2	(22%)	1	(6%)
4–6 years	7	(27%)	2	(22%)	5	(29%)
7–10 years	6	(23%)	2	(22%)	4	(24%)
>10 years	10	(38%)	3	(33%)	7	(41%)
Familiarity with results: n (%^c^)						
Some familiarity	11	(42%)	8	(89%)	3	(18%)
Familiar and have read the paper	15	(58%)	1	(11%)	14	(82%)
Reported treatment difference at 3 months: n (%^c^)						
EVAR QoL > OPEN QoL	21	(81%)	7	(78%)	14	(82%)
OPEN QoL > EVAR QoL	1	(4%)	1	(11%)	0	(0%)
No difference	2	(8%)	0	(0%)	2	(12%)
Not sure	2	(8%)	1	(11%)	1	(6%)

a Includes vascular nurse specialists, research nurses and a consultant vascular nurse.

b Includes consultant vascular surgeons, a consultant interventional radiologist and a vascular academic junior doctor acting as site trial coordinator.

c Percentage of column total.

Table 2 reports the elicitation responses. Overall, for a typical patient in the OPEN arm, the elicited QoL scores were lower versus the corresponding average score from the observed data, with a mean difference of four units on the 0–100 scale. For patients with missing QoL, the mean elicited values were on average 11 units higher for patients in the eEVAR versus Open repair arms. In general, the nurses tended to be more optimistic than the doctors about the expected outcomes of OPEN patients (with incomplete QoL data), but there was less difference in their views about the eEVAR patients. Half the experts believed the QoL scores for non-respondents in the eEVAR and OPEN arms were positively correlated, and all except one of the others reported no correlation. The supplementary material contains more detail.

**Table 2.** Summary of elicited QoL scores for IMPROVE trial patients with missing versus observed data.

	All	Nurses^a^	Doctors^b^
Number of responses^c^	25	8	17
Elicited scores: mean (SD)			
Typical OPEN arm patient, who did not return a completed QoL questionnaire			
Most likely QoL score (mean of normal distribution)	61 (17)	71 (10)	56 (17)
Uncertainty about QoL score (SD of normal distribution)	16 (12)	18 (14)	15 (11)
Typical eEVAR arm patient, who did not return a completed QoL questionnaire			
Most likely QoL score (mean of normal distribution)	72 (15)	76 (11)	70 (17)
Uncertainty about QoL score (SD of normal distribution)	15 (12)	18 (13)	14 (12)
Differences in scores: mean (SD)			
Typical OPEN arm patients, did not return QoL – did return QoL	−4 (17)	6 (10)	−9 (17)
Typical did not return QoL patients, eEVAR arm – OPEN arm	11 (11)	4 (12)	14 (10)
Correlation between QoL scores for non-respondents in the eEVAR and OPEN arms n(%^d^)			
Positive	13 (52%)	3 (38%)	10 (59%)
Zero	11 (44%)	5 (62%)	6 (35%)
Negative	1(4%)	0(0%)	1(6%)

SD: standard deviation; eEVAR: emergency endovascular strategy; QoL: quality of life.

The QoL scale is from −20 to 100, and mean (SD) is across experts.

a Includes vascular nurse specialists, research nurses and a consultant vascular nurse.

b Includes consultant vascular surgeons, a consultant interventional radiologist and a vascular academic junior doctor acting as site trial coordinator.

c Excludes one nurse who expressed almost complete uncertainty about the quality of life scores.

d Percentage of column total.

**Figure 3.**
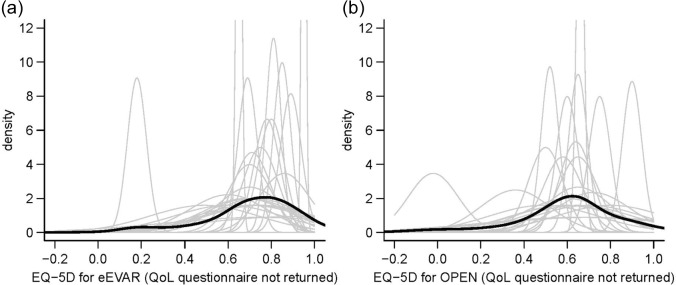
Individual and pooled prior distributions for patients randomised to eEVAR and open repair arms: (a) eEVAR: all experts and (b) OPEN: all experts. Thin grey lines = individual priors, thick black lines = smoothed pooled priors across all experts. Although each individual prior has been elicited as a normal distribution, this restriction does not apply to the pooled priors which are a mixture of normal distributions.

As Figure 3 shows, for both trial arms, there is a wide diversity across the experts in the elicited QoL scores for patients with missing data. The bold black lines indicate the result of combining the views of all the experts in each trial arm using linear pooling.

### Implications for the effectiveness of eEVAR versus OPEN

**Figure 4.**
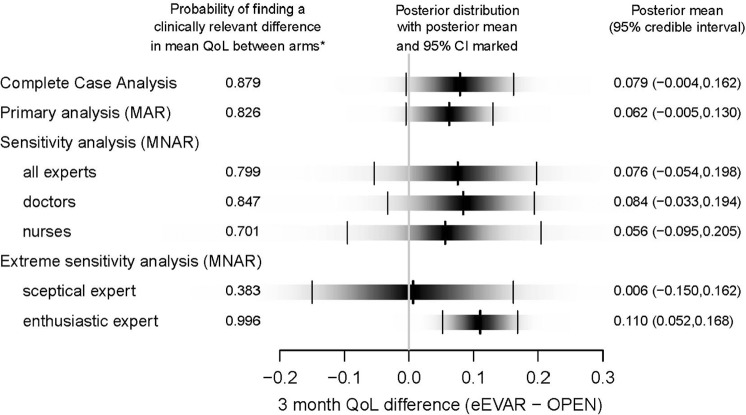
Difference in mean quality of life score at 3 months between randomised arm (eEVAR - open repair) for survivors. Each shaded rectangular strip shows the full posterior distribution of the difference in mean QoL at 3 months for survivors for one model run. The darkness at a point is proportional to the probability density, such that the strip is darkest at the maximum density and fades into the background at the minimum density. The posterior mean and 95% credible interval are marked. *the posterior probability that the eEVAR QoL at 3 months is at least 0.03 greater than the open repair QoL. 0.03 is the minimum clinically important difference.^31^

The results of our sensitivity analysis compared to the complete case and MAR analyses are reported in Figure 4 as (1) the posterior probability that the eEVAR QoL at 3 months is at least 0.03 units greater than the OPEN QoL, where 0.03 is the minimum clinically important difference,^31^ alongside (2) the posterior distribution of the difference in the mean 3-month QoL scores between the two arms. The full posterior distribution is shown as a density strip, where the darkness at a point is proportional to the probability density.^32^

The estimated effect of randomised arm on average QoL score is generally similar across the alternative approaches to the missing data, but the sensitivity analysis resulted in substantially greater uncertainty about this mean difference. That is, the credible intervals from the MNAR are wider than following the MAR and complete case analyses. These wider credible intervals recognise the variation within and across experts in the likely differences in outcomes for patients with missing versus observed QoL data. The doctor and nurse subgroup results are broadly similar to the overall result. However, the extreme individual priors give markedly different results and levels of uncertainty, for the ‘optimistic’ expert the probability of a clinically important difference in favour of eEVAR is 100% while the corresponding probability for the ‘sceptical’ expert is 38%.

## Discussion

We successfully developed and demonstrated a user-friendly tool for eliciting the expert opinion required for recommended sensitivity analysis for missing data. The tool uses existing open source software and can be administered face-to-face or online, to elicit beliefs from reasonably large numbers of experts without imposing an undue burden. We have shown that the elicited views can be converted into informative priors for the sensitivity parameters in a pattern-mixture model, allowing for correlation in the elicited values across the trial arms. Trial data can then be re-analysed under different MNAR assumptions to explore the robustness of the results.

This article contributes to the literature in several ways. First, by providing a practical tool that can quickly elicit the views of a range of experts, this research will help make recommended approaches to sensitivity analyses accessible to a wide range of trial settings. Second, the new tool goes further than those developed previously, eliciting expert views about the correlation between the randomised arms in the outcomes for those with missing data. Third, the article contributes to knowledge about the relative effectiveness of a potentially important intervention eEVAR versus open repair for patients with ruptured aortic aneurysm. The sensitivity analysis builds on the previously published research in finding that eEVAR does increase the mean QoL at 3 months post-randomisation even after recognising that data may be MNAR.

A key reason that our elicitation exercise was successful was because it was undertaken alongside an active trial and annual society meeting for the clinicians involved, which allowed for ease of access to experts with the requisite knowledge about the patients with missing data. Also, the study was carefully designed to focus the elicitation exercise on gaining expert opinion on the key parameters required to avoid creating an exercise that was too burdensome. The qualitative questions allowed assessment of the experts’ engagement in the exercise, indicated a general consensus that eEVAR patients recovered more rapidly and provided reasons for the missing outcome data according to unobserved aspects that were therefore not accounted for in the MAR analysis. These included degree of physical and psychological recovery, personality of patient, lack of family support, financial pressures, family bereavement, social life, dislike of paperwork, forgetfulness, loss of interest in the study and lack of appreciation of the importance of completing the questionnaire.

Our tool has been designed to be generally applicable to RCTs with different designs and with alternative endpoints and can be extended in several ways. In IMPROVE as in other studies, there is interest in whether the treatment effect is modified by subgroup, in this case according to age, gender and the Hardman index which measures the patients’ baseline severity. A potential extension would be to elicit expert beliefs on the differences in average outcomes for the different patient subgroups. Similarly, in IMPROVE, as in many technology assessments, there is interest in the long-term effectiveness of the intervention. While the experts offered the view that the gain in QoL for eEVAR versus open repair would be maintained at 12 months post-randomisation, it would be helpful to extend the elicitation exercise to inform sensitivity analyses at multiple time points.

Our proposed elicitation tool is generalisable to other clinical trials by adapting both the set of questions and response options. To encourage methods uptake, R code for implementing the expert elicitation is available in the online supplementary material. Furthermore, the priors elicited from such primary research, as undertaken alongside the IMPROVE trial, could be ‘borrowed’ by future studies of the same intervention (e.g. other eEVAR trials) to explore the robustness of their conclusions. Potentially, a series of reference priors for different disease areas could be developed to facilitate MNAR sensitivity analysis without undertaking primary elicitation exercises.

The approach could be used at the design stage, utilising either previously collected priors or new priors elicited from the trial team. Combining these with the expected level of loss to follow-up could provide an improved estimate of the likely impact of missing data on the trial’s results. Hence, this approach could help improve trial design, so that the study results are more robust to anticipated levels of missing data.

An alternative approach is double sampling,^33–35^ which seeks to collect additional information from those whose data are missing. The validity of this approach depends on the (often untestable) assumption of outcome stability, and re-contacting patients may raise ethical and practical issues. However, in trials where the main concern is in missing data for outcomes other than QoL, where the assumption of outcome stability is more plausible, it would be interesting to contrast the results of double sampling with expert elicitation.

### Limitations

A potential limitation with sending by email is that there might be compatibility issues between Rshiny and older versions of web browsers. The flexibility of administering the tool face-to-face helps address this drawback.

Our elicitation tool is intended to be widely accessible to clinical investigators, and to achieve this goal, we made several simplifying assumptions. First, we assumed that individual expert opinion could be adequately represented as a normal distribution. The experts at the pilot stage considered this assumption reasonable, and only two experts contributing to the main elicitation indicated that the normal distribution was restrictive. More generally, the elicitation literature suggests that it may be preferable to avoid imposing a parametric distribution that artificially constrains beliefs.^36^ In future, we therefore plan to extend our elicitation tool to allow greater flexibility in the distribution of possible values. This restriction does not apply to the priors used in our models, for example, the pooled priors are a mixture of normal distributions. Second, the elicitation exercise was undertaken after the primary analysis concerning the outcome of interest, QoL at 3 months post-randomisation, had been published. Inevitably, experts’ priors are informed by prior evidence and knowledge of the results of the trial in question, and related evidence and this may influence their views. Here, we found little difference between the subgroup of experts who were aware of the published results versus those who were not. Nevertheless, we recommend that this type of elicitation is carried out before the trial results are known if at all possible. When this is not possible, it is important that the analysis investigates the likely implications for interpretation of the results.

Future versions of the tool should improve on the wording describing the scenarios. In particular, in response to a reviewer’s suggestion, we recommend altering the introductory sentences to the main questions, to sharpen the distinction between the variance of an observation and the variance of a mean. For example, before future use of the tool in Question 2, we would replace the preamble wording (‘the range of values which you believe are plausible’) by the more precise, correct, wording used in asking the question (‘your opinion of the most likely quality of life score for Bill’). We anticipate that users of the tool will need to modify the text and images. Such modifications need to maintain this distinction.

## Summary

This article successfully demonstrates a general and practical approach, for eliciting expert opinion and conducting sensitivity analysis to assumptions about missing data in clinical trials.
